# Early Increase and Late Decrease of Purkinje Cell Dendritic Spine Density in Prion-Infected Organotypic Mouse Cerebellar Cultures

**DOI:** 10.1371/journal.pone.0081776

**Published:** 2013-12-02

**Authors:** Jody L. Campeau, Gengshu Wu, John R. Bell, Jay Rasmussen, Valerie L. Sim

**Affiliations:** 1 Centre for Prions and Protein Folding Diseases, University of Alberta, Edmonton, Canada; 2 Department of Medicine (Neurology), University of Alberta, Edmonton, Canada; 3 Centre for Neuroscience, University of Alberta, Edmonton, Canada; University of Maryland School of Medicine, United States of America

## Abstract

Prion diseases are infectious neurodegenerative diseases associated with the accumulation of protease-resistant prion protein, neuronal loss, spongiform change and astrogliosis. In the mouse model, the loss of dendritic spines is one of the earliest pathological changes observed *in vivo*, occurring 4–5 weeks after the first detection of protease-resistant prion protein in the brain. While there are cell culture models of prion infection, most do not recapitulate the neuropathology seen *in vivo*. Only the recently developed prion organotypic slice culture assay has been reported to undergo neuronal loss and the development of some aspects of prion pathology, namely small vacuolar degeneration and tubulovesicular bodies. Given the rapid replication of prions in this system, with protease-resistant prion protein detectable by 21 days, we investigated whether the dendritic spine loss and altered dendritic morphology seen in prion disease might also develop within the lifetime of this culture system. Indeed, six weeks after first detection of protease-resistant prion protein in tga20 mouse cerebellar slice cultures infected with RML prion strain, we found a statistically significant loss of Purkinje cell dendritic spines and altered dendritic morphology in infected cultures, analogous to that seen *in vivo*. In addition, we found a transient but statistically significant increase in Purkinje cell dendritic spine density during infection, at the time when protease-resistant prion protein was first detectable in culture. Our findings support the use of this slice culture system as one which recapitulates prion disease pathology and one which may facilitate study of the earliest stages of prion disease pathogenesis.

## Introduction

Prion diseases are fatal neurodegenerative diseases of humans (Creutzfeldt-Jakob disease (CJD)), cattle (bovine spongiform encephalopathy (BSE)), cervids (chronic wasting disease (CWD)) and sheep (scrapie). They arise when the normally expressed alpha-helical prion protein (PrP^C^) misfolds and aggregates as a beta sheet-rich form (PrP^Sc^) [1], [2]. PrP^Sc^ then seeds the conversion of more PrP^C^ into PrP^Sc^, leading to the hallmark neuropathology of prion diseases, PrP^Sc^ accumulation, neuronal loss, spongiform change and astrogliosis. The earliest pathological changes, occurring before neuronal loss, involve neuronal synapses, specifically the loss of dendritic spines and a concomitant loss of long term potentiation [3]–[7]. These changes have been observed in animal models of prion infection, but not in cell culture models of prion infection.

Organotypic cultures have advantages over simple cell cultures in that they largely recapitulate the *in vivo* microenvironment [8], [9] while providing a system which is more open to experimental manipulation than the whole animal. The prion organotypic slice culture assay [10], [11] (POSCA) was developed as an assay for the detection and *ex vivo* replication of prion infectivity, using a slice culture from a neonatal mouse cerebellum. These cultures can be infected with a number of different prion strains, can be maintained for many weeks, and can generate and accumulate PrP^Sc^ at a much faster rate than *in vivo*, with PrP^Sc^ detectable within 21 days. However, the fact that a cultured brain slice can replicate PrP^Sc^ does not *de facto* indicate that prion infection –specific pathological changes will also develop within that slice. One study has reported some prion-specific neuropathological changes detectable by electron microscopy in these infected cultures, specifically the development of small vacuolar degeneration and tubulovesicular bodies [12], but to our knowledge, quantification of dendritic spine density has not been performed.

While the development of dendritic varicosities and loss of dendritic spines does co-localize to areas of vacuolar and prion protein pathology [13], spine loss in hippocampal neurons *in vivo* is not observed until 4–5 weeks after the initial detection of PrP^Sc^ at day 70 post-inoculation [3]. Given the accelerated replication of PrP^Sc^ in POSCA, detectable by day 21, we hypothesized that spine loss might also be accelerated, and thus detectable within the lifespan of the culture. Within the cerebellum, Purkinje cells are readily identifiable by post-natal day ten and have elaborate dendritic trees amenable to analysis; they are also known to undergo dendritic disintegration *in vivo* when infected by prion strains targeting the cerebellum [14]. As such, we chose to analyze Purkinje cell dendritic spine density over the course of prion infection in POSCA.

We demonstrate that Purkinje cell dendritic spine loss does occur in POSCA, within a timeframe similar to pathogenesis *in vivo*. In addition, we found that Purkinje cell spine density increased transiently during early infection, at the time PrP^Sc^ was first detectable.

## Materials and Methods

### Ethics statement

This study was carried out in strict accordance with the recommendations in the Canadian Council on Animal Care, as approved by the Animal Care and Use Committee (ACUC) of the University of Alberta (Study ID AUP00000335; Animal Welfare Assurance Number: #A5070-01). Mice were bred and pups were used as source material for cultures. All mice were bred in an ACUC approved facility with environmental enrichment. Any animal showing signs of distress, including loss of more than 20% body weight, were euthanized by CO2 asphyxiation. Mouse pups were rapidly euthanized by cervical dislocation to provide healthy brain tissue for culture.

#### 1) Prion Cerebellar Organotypic Slice Culture Assay

Experiments were performed as previously described (Falsig et al. 2008) on 11 day old tga20/+ mice on a C57BL6 background [15] (kindly provided by Dr. Frank Jirik, University of Calgary). In summary, mice were euthanized and cerebella were removed and embedded in ultralow-melting-point agarose (Invitrogen) dissolved in GBSSK (Grey's balanced salt solution, 1 mM kynurenic acid, 33.3 mM D-glucose). 350 µm thick sagittal slices were cut on a Vibratome 1500 and stored 1.5–6 hours until use in 15 mL ice cold GBSSK. Slices from each animal (10–16 per animal) were plated into a 6-well plate using sterilized blunt ended 1000 µL pipette tips into 1.0 mL GBSSK. Cultures were treated with 1.0 µg/mL RML prion-infected or uninfected brain homogenates (homogenized in PBS) diluted in 1 mL GBSSK. Tissues were incubated with brain homogenates as free-floating sections for 1 h on ice and washed three times in 1.0 mL ice cold GBSSK. Two to three slices were placed on each 12 mm Millicell PTFE 0.4 µM membrane cell culture insert (Millipore). Residual GBSSK was removed from the apical and basolateral compartment of the well. Slices were cultured with 200 µl of warm (37°C) slice culture medium (50% vol/vol MEM, 25% vol/vol basal medium Eagle and 25% vol/vol horse serum supplemented with 0.65% glucose (wt/vol), penicillin/streptomycin and Glutamax (Invitrogen) added the basolateral compartment of the well. Cultures were kept in a standard cell incubator (37°C, 5% CO_2_ and 95% humidity) and all of the culture medium was exchanged three times a week. Each mouse pup produced five to six wells and samples from each mouse pup were treated as separate biological replicates. Weekly, a single insert from each animal was used for detection of PrP^Sc^ by western blotting or for immunofluorescent staining and analysis of dendritic spines. Cell death was monitored by Toxilight assay (Lonza), propidium iodide staining, or TUNEL staining.

#### 2) Immunofluorescence and microscopy

From each mouse pup source, a single insert with 2–3 cerebellar slices was taken weekly for analysis. For the dendritic analysis experiments, a total of sixteen mice were used for culture preparation, eight sets infected, eights sets uninfected. Half the pup samples were imaged on days 7, 14, 21, 28, 35, 42, and the other half were imaged on days, 35, 42, 49, 56, 63, and 70 post-infection.

At selected time points, slices on their inserts were stained with 10 µg/mL propidium iodide (PI) (Invitrogen) in warm medium for 15 min, washed ×3, fixed with fresh 4% PFA (Invitrogen) for 15 min, washed ×3, permeabilized with 0.25% tritonX-100 for 15 min, washed ×3, blocked with 1% Goat serum/1% BSA for 1 hr, treated with 1∶4000 anti-mouse calbindin (AB Cam) in blocking buffer for 1 hr, washed ×3, treated with 1∶4000 anti-goat Alexa Fluor 488 (Invitrogen) for 30 min, and washed ×3. Dead nuclei were quantified in all images used for analysis, at 63× magnification. The number of degenerating nuclei positive for PI staining were tallied using Imaris software, and this number was divided by the number of total nuclei (DAPI positive), to achieve a ratio of dying to healthy cells for each image. Statistical significance was determined by an unpaired, 2 tailed t-test assuming equal variance.

For the Tunel Assay, the Deadend™ Fluorometric Tunnel assay (Promega) was used. Slices on inserts were fixed in 4% PFA (Invitrogen) in PBS for 25 min at 4°C, washed ×3, permeabilized with 0.25% tritonX-100 at RT for 10 min, washed ×3, equilibrated in equilibration buffer for 10 min, then treated with rTdT incubation buffer at 37°C for 1 hr in a humidified chamber to allow tailing reaction to occur. The reaction was stopped using 2XSSC at RT for 15 min and slices were washed ×3. Then immunofluorescence labelling proceeded from the blocking step as described as above.

All washes were performed apically and basolaterally with PBS pH 7.4. All stains were prepared in PBS pH 7.4. After labelling, membranes were removed from the insert support and placed on a slide with the apical surface of the tissue up. 3 drops of Prolong gold with DAPI (Invitrogen) were placed on the membrane insert and a coverslip was affixed to the slide. Slides were cured a minimum of 24 hrs.

Sixteen bit z-stacked images were acquired with a Zeiss LSM 700, 63x, oil objective, using Nyquest sampling for a final resolution of 0.07 µm in xy and 0.38 µm in z axes.

#### 3) Western blotting

To detect PrP^Sc^, single inserts with 2–3 cerebellar slices were rinsed 2x, then homogenized in 25 µL ice cold RIPA buffer. Total protein concentration was determined by BCA assay. 30 µg total protein (in 20 µL) was treated with 20 µg/mL Proteinase K (Invitrogen) at 37°C for 1 hr, diluted to 33.3 µL with PefaBloc (1 mM) and 3X loading buffer (187.5 mM Tris HCl pH 6.8, 15% glycerol, 15% SDS, 9 mM EDTA, 8 M urea, 12% βME), boiled for 10 min, and stored at −80°C. A 5 µL portion was diluted to 100 µL with 1X RIPA, PefaBloc (1 mM) and 3X loading buffer, boiled for 10 min, and stored at −80°C. PK-treated samples were loaded at 10X higher concentration than the non-PK treated samples. Proteins were separated on a Novex 4%–12% Bis-Tris polyacrylamide gel (NuPAGE, Invitrogen) and blotted onto a PVDF membrane, blocked with 5% milk in Tris-buffered saline supplemented with Tween (TBST) (150 mM NaCl, 10 mM Tris HCl, 0.05% Tween 20 (vol/vol). Mouse PrP was detected using SAF83 antibody (1∶20,000 Cayman chemical) in 5% milk TBST. Secondary antibodies used were Alkaline phosphatase tagged anti mouse IgG (Promega). Blots were developed using AttoPhos substrate (Promega) and detected on an Image quant LAS 4000 (GE Healthcare).

#### 4) Dendritic spine analysis

Confocal images were deconvolved using ImageQuant X, and spines were identified and quantified using Imaris 7.1.1 software. The images were taken of each slice. For each image, three representative dendritic trees were chosen for analysis and from each of these, three terminal branch points were analyzed. Variability between measurements was not influenced by the pup used as culture source, so all measurements were averaged in the final analysis. Statistical significance was determined by an unpaired, 2 tailed t-test assuming equal variance. Only dendrites approximating the horizontal plane of the slice were chosen for analysis, taking advantage of optimal resolution in the xy plane (0.07 µm in xy and 0.38 µm in z axes).

## Results

### PrP^Sc^ production

**Figure 1:**
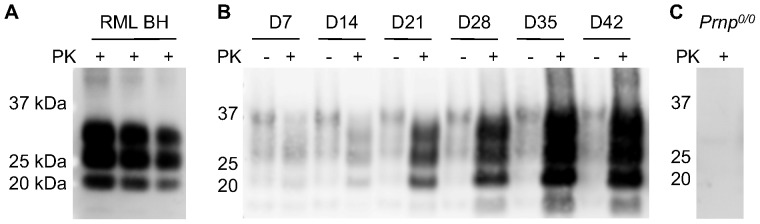
Immunoblots of PrP^Sc^ in cerebellar slice cultures infected with RML strain. A) The typical banding pattern of RML-infected brain homogenate (RML BH) inoculum after proteinase K (PK) digestion is shown. 10, 5, and 2 µg total protein were loaded. B) RML-infected POSCA slices were homogenized in pairs in RIPA buffer, then 30 µg total protein was treated with 20 µg/mL PK for one hour (+). Undigested slice culture homogenates (−) were loaded at 10x lower concentration for comparison. PrP^Sc^ is distinguished by its truncated state after PK digestion, seen as lower molecular weight bands when compared with PrP^C^. PrP^Sc^ can also been seen to increase in amount with increasing culture time (days 7 through 42 are shown). C) RML-treated POSCA slices from *Prnp^0/0^* mice did not show PrP^Sc^ at any time point. Day 45 is shown.

We prepared cerebellar slice cultures from 11 day old tga20 mouse pups and infected them with RML strain prions as previously described [10], [11]. At weekly time points, samples were homogenized and treated with proteinase K to digest PrP^C^ and leave only PrP^Sc^ detectable by Western blot. The banding pattern of PrP^Sc^ produced by the cultures did not differ from that of the inoculum (Fig 1A) and increased in amount over time (Fig 1B), consistent with ongoing infection. The PrP^Sc^ produced in these cultures was also able to reinfect subsequent cultures (data not shown). Control slices treated with uninfected brain homogenate or PrP knockout slice cultures exposed to infected brain homogenate did not generate any PrP^Sc^ (Fig 1C).

### Dendritic spine analysis

**Figure 2:**
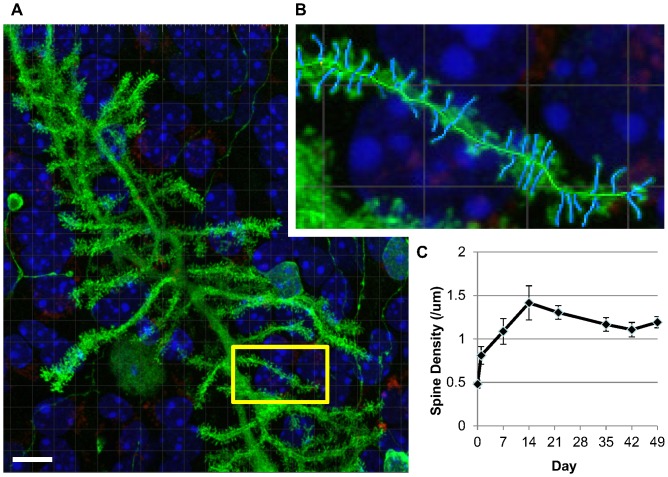
Purkinje cell spine density analysis; stability in uninfected cerebellar slice cultures from C57Bl6 mice. A,B) Representative Z-stacked image of a Purkinje cell as analyzed with Imaris 7.1.1 software, after deconvolution with ImageQuant X. B) Inset of A showing dendritic spine analysis; spines identified in blue. Nuclei are stained with Hoechst (blue), dead cells are stained with propidium iodide (red) and Purkinje cells are labelled with calbindin/Alexafluor488 (green). Scale bar 10 µm. C) Average spine density from uninfected cerebellar cultures from C57Bl6 mice at weekly time points. Errors bars represent one standard error of the mean. n = 9 except day 14 where n = 3.

The mouse cerebellum is mostly developed by postnatal day 10 with established neuronal projections [8], [9], [16], but we sought to confirm the stability and variability of Purkinje cell dendritic spine densities in long-term cerebellar slice cultures prepared from day 10–12 mouse pups. We initially prepared uninfected cerebellar slice cultures from uninfected wild type C57Bl6 mice. We cultured slices for 49 days and, at weekly intervals, collected slices for immunolabeling, confocal imaging and dendritic spine quantification. On the day of culture preparation (day 0), spine densities in the slices were lowest (0.48±0.12 per µm) but steadily increased until day 14, after which they remained at an average of 1.2 spines per µm with an average standard deviation of 0.24 per µm (Fig 2).

Having demonstrated the technical feasibility of spine assessment and the stability of spine densities over time, we prepared slice cultures using the tga20 mice as described in the original POSCA protocol [10], [11], exposing them to RML-infected brain homogenate or normal brain homogenate. Tga20 mice do not develop spontaneous disease but express 5–6 times more PrP^C^ than C57Bl6 mice and thus develop prion disease faster. We therefore predicted that any pathology that might develop in POSCA would also occur more rapidly in the tga20 mice.

**Figure 3:**
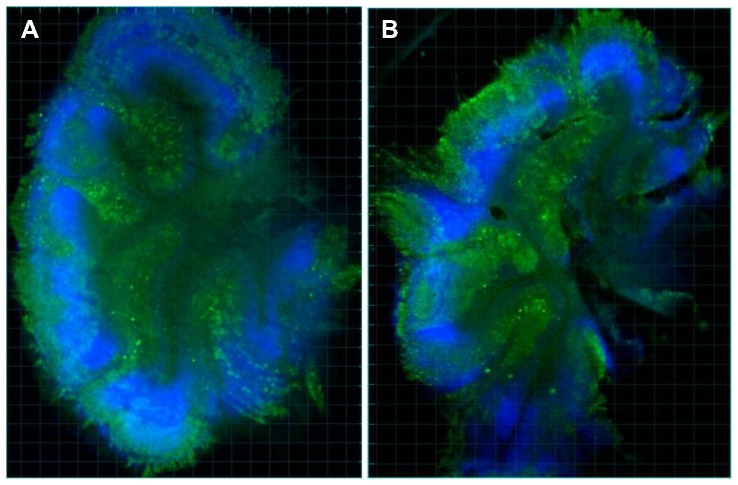
Cerebellar slice architecture after 42 days of culture. Low resolution images (2X) of uninfected (A) and infected (B) cerebellar slices at day 42, demonstrating preserved cerebellar architecture and Purkinje cells. Imaged on an InCell Analyzer. Nuclei are stained with Hoechst (blue) and Purkinje cells are labelled with Calbindin/Alexafluor488 (green).

Slices were cultured up to 69 days, with samples collected for imaging at weekly intervals. During the culture period, slices became thinner (originally 350 µm thick), but the architectural definition of the molecular, Purkinje cell and granular layers remained distinct (Fig 3). We were able to image Purkinje cells, after fixation and Calbindin labelling, within both the infected and uninfected cultures at weekly time points. Data for infected and uninfected slices was analyzed both by comparing average spine densities from slices derived from individual mouse pups, and by averaging all spine densities, treating each measurement as an individual replicate. Variability between mouse pups was not statistically different from variability between individual slice measurements, so each measurement was treated as an independent replicate.

**Figure 4:**
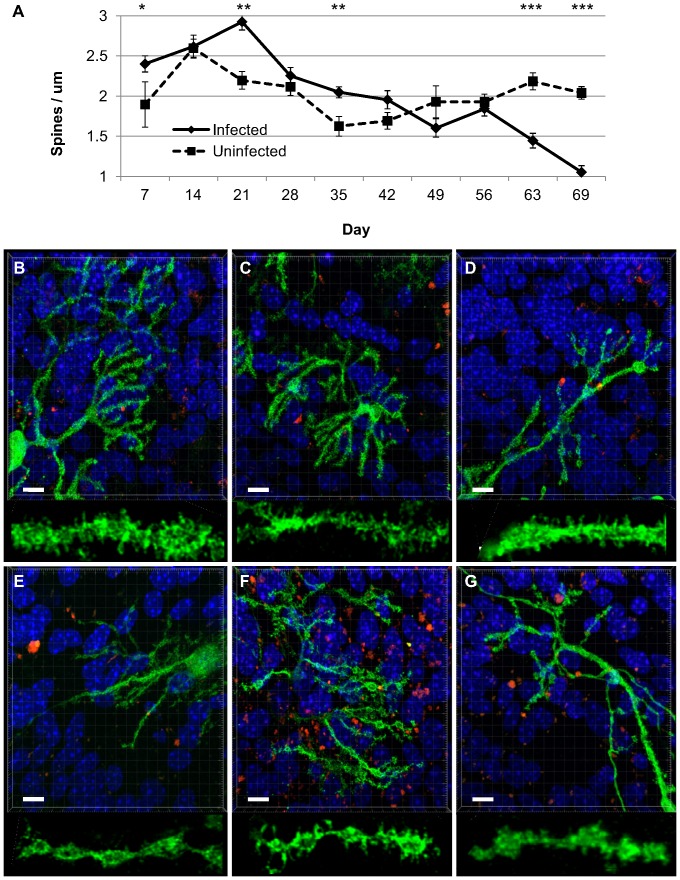
Purkinje cell dendritic spine densities in RML-infected and uninfected cerebellar slice cultures. A) Average Purkinje cell spine densities at weekly time points. B–G) Representative images from uninfected (B–D) and RML-infected (E–G) cultures at day 69. Nuclei are stained with Hoechst (blue), dead cells are stained with propidium iodide (red) and Purkinje cells are labelled with Calbindin/Alexafluor488 (green). Scale bar 10 µm. Insets below each panel are magnified by a factor of three to better visualize dendritic spines. Images of fixed samples were taken on a Zeiss LSM 700, 63x oil objective, deconvolved with ImageQuant X and analyzed using Imaris 7.1.1 software. The average n was 23 per time point for infected (range 12–41), 14 for uninfected (range 6–22). At day 69, n = 17 for infected, n = 21 for uninfected. p-values: * <0.05; ** <0.01; *** <0.001.

As expected from studies in wild type mice, spine densities in uninfected tga20 cultures remained stable after day 14 (Fig 4A). Interestingly, the average spine densities from tga20 cultures after day 14 were 2.0 per µm, which is significantly higher than the 1.2 per µm seen in the C57Bl6 cultures. Spine densities remained stable in the uninfected cultures; in infected cultures, dendritic spines were reduced after 63 days of culture (p<0.001). By this late stage of culture, fewer Purkinje cells remained in both the infected and uninfected cultures, but in the uninfected cultures, Purkinje cells retained spine densities and dendritic morphologies whereas those in the infected cultures had fewer spines and dendritic morphology was clearly altered (Fig 4B–G). For all images used in analysis, the ratio of degenerating nuclei stained with PI was divided by the number of normal nuclei stained with DAPI, to determine the amount of cell death. The number of dying cells was not statistically different between infected and uninfected slices at any time point, although we cannot account for any dead cells on the surface of the slices that may have been washed off during wash steps.

### Early changes in synapse markers

**Figure 5:**
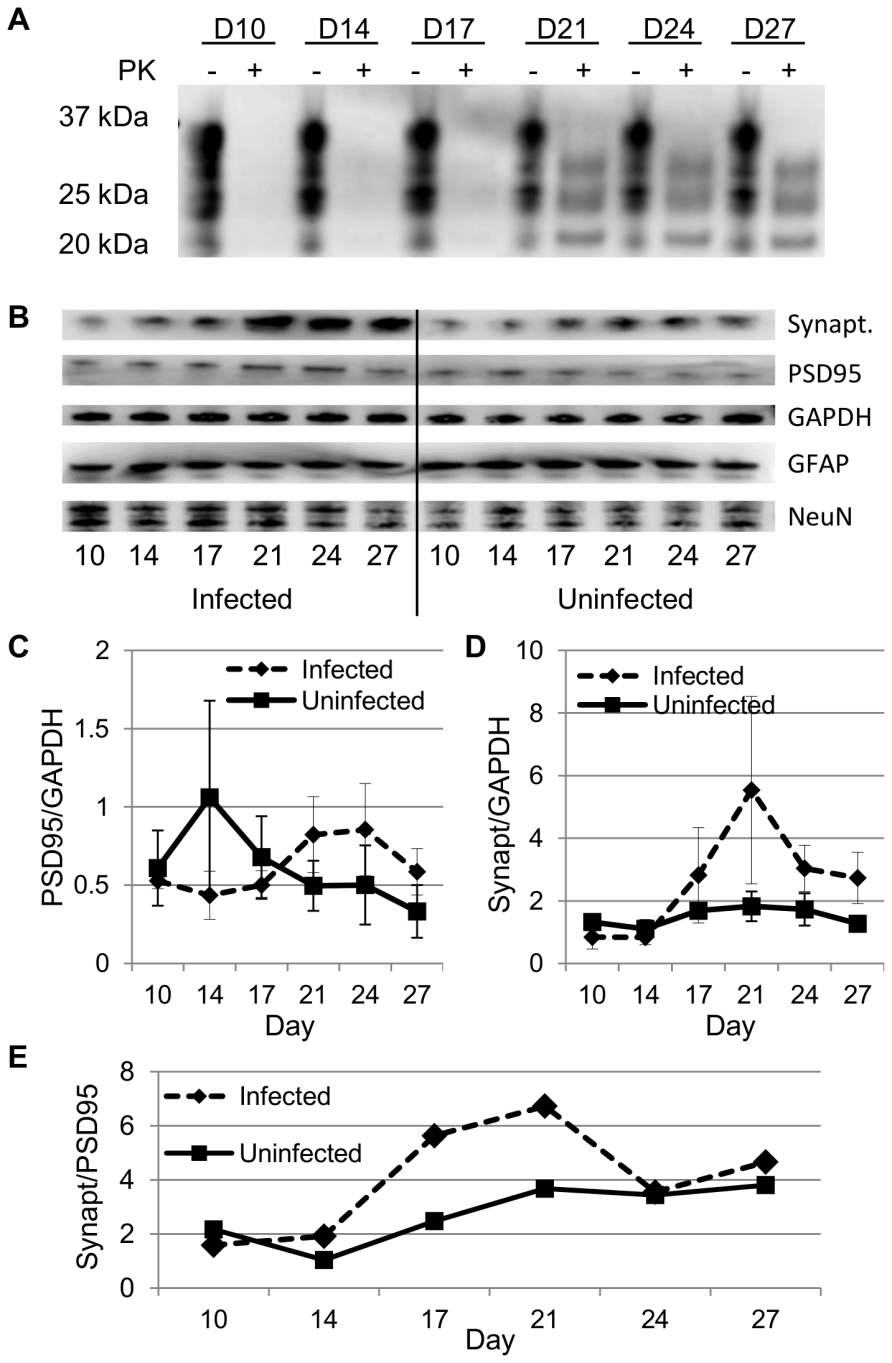
Immunoblots of RML-infected and uninfected cerebellar slice cultures from tga20 mice at early time points. A) Representative immunoblot of PrP^Sc^ in infected slice cultures before (−) and after (+) proteinase K (PK) digestion. IPC1 antibody was the primary antibody. 4 of 5 experiments first detected PrP^Sc^ at day 21, 1 was detected by day 17. Undigested samples were diluted by 10x before loading. B) 2–3 slices were collected at days 10 through 27, normalized for total protein by BCA assay, and probed for post-synaptic density 95 (PSD95), pre-synaptic synaptophysin (synapt.), GAPDH (protein loading control), GFAP (astrocyte marker) and NeuN (neuronal marker). C) Levels of PSD95, normalized to GAPDH, in infected and uninfected cultures at weekly time points. Differences were not statistically significant. D) Levels of synaptophysin, normalized to GAPDH, in infected and uninfected cultures at weekly time points. Differences were not statistically significant. E) Ratios of GAPDH-normalized synaptophysin/PSD95 are shown for infected and uninfected cultures at weekly time points. Each data point represents a single ratio from averages from all experiments.

There were lesser, but statistically significant, differences in spine densities at other time points (Fig 4A). Unlike the reduced spine density at day 63 and 69, spine densities at day 7, 21, and 35 were actually higher in the infected slices. Dendritic morphologies were not altered at these time points. The greatest difference was noted at day 21; infected cultures had spine densities of 2.9±0.6/µm whereas uninfected slices were 2.2±0.3/µm (p<0.01). Interestingly, this timing corresponded to the first detection of PrP^Sc^ in the cultures. To confirm whether this increase in Purkinje cell spine density reflected overall changes in synapse protein levels throughout the slice at these early time periods, we repeated infection experiments focussing on early time points, collecting samples for immunoblot every 3–4 days starting at day 10. In four of five replicate experiments, PrP^Sc^ was first detected at day 21 (Fig 5A), in one of the five it was detectable by day 17. Two slices from each experiment were pooled at a given time point, normalized for total protein content prior to loading and immunoblotted for post-synaptic density 95 (PSD95), pre-synaptic synaptophysin, GFAP (astrocyte marker), NeuN (neuronal marker), and/or GAPDH (protein loading control) (Fig 5B). Within individual experiments, PSD95 and synaptophysin levels were always higher in infected slices by day 21 compared with uninfected slices, but this did not reach statistical significance (Fig 5C,D). Interestingly, the ratio of synaptophysin to PSD95 increased from 1.0 to 3.8 in uninfected cultures, but peaked at 5.6 and 6.7 at days 17 and 21, respectively, in infected cultures (Fig 5E).

## Discussion

Dendritic spines are sites of synapse contact from other neuronal axons. They are critical to processes of memory and learning, undergoing long term potentiation (LTP) or depression (LTD) to reinforce or reduce synaptic pathways, respectively [17]. In prion disease, the loss of dendritic spines is one of the first observable pathological signs [3]–[7] and there is a corresponding reduction in LTP [5]–[7]. There is controversy as to whether PrP^Sc^ localizes to the pre-synaptic areas [18] or to the dendrites of neurons [19], but dendritic spine loss does co-localize to areas of vacuolar and prion protein pathology [13]. Purkinje cells have also been observed to undergo dendritic disintegration *in vivo* when infected by prion strains targeting the cerebellum [14]. Using the cerebellar slice culture model of prion disease (POSCA) introduced by Falsig et al. [10], [11], we have demonstrated that Purkinje cell dendrites are vulnerable to spine loss and morphological alteration after infection with RML prions.

*In vivo*, the development of dendritic varicosities and the loss of dendritic spines has been observed 4–5 weeks after the initial detection of PrP^Sc^ at day 70 post-inoculation [3]. Given the detection of PrP^Sc^ in POSCA by 21 days post-infection, we predicted that spine loss might be detectable within the lifetime of the culture. Indeed, significant spine loss and morphological change was observed after 63 days of culture, 6 weeks after initial PrP^Sc^ detection. While this could imply that spine loss takes longer to occur in culture than *in vivo*, the *in vivo* data is derived from cortical and hippocampal neurons, not Purkinje cells, so a direct comparison cannot be made. In addition, the RML prion strain does not generate a high level of PrP^Sc^ in the cerebellum *in vivo*; other prion strains such as 22L, which is known to cause more pathology in the cerebellum, may produce more significant or earlier dendritic spine changes in cerebellar culture. Regardless of the exact timing of spine loss in POSCA relative to that which occurs *in vivo*, spine loss does occur in a timeframe very similar to that seen in mouse models; our findings support the argument that this model system recapitulates at least some aspects of prion disease pathogenesis, not just prion replication, and as such is an extremely valuable tool both for studying prion disease pathogenesis as well as for screening treatments.

During our analysis of spine densities in POSCA, we incidentally discovered an increase in Purkinje cell spine density in infected cultures, occurring within weeks of infection. After analyzing synapse protein levels in the whole cerebellar culture, a trend towards increases in synaptophysin and PSD95 was also seen. This increase coincided with the first detection of PrP^Sc^ by Western blot analysis. Of particular note is the greater increase in synaptophysin levels when compared with PSD95. These were small changes that did not reach statistical significance but they could suggest that presynaptic stimulation is occurring. While this may be a non-specific response by the tissue in culture, it was only observed in infected slices and there is some evidence that a similar phenomenon may be occurring *in vivo*. Transient elevations of kinases which promote dendritic spine development, including EphA4, CaMK4β, and PKG1, have been detected through kinomic analyses of samples from wild type mice infected with RML prion isolates. These elevations are noted at approximately 70 days post-inoculation, which is the approximate time at which PrP^Sc^ is first detectable in these models [20]. In a tg37 mouse model, synaptic proteins PSD95 and SNAP25 are increased a week prior to and at the time of PrP^Sc^ detection by immunohistochemistry [21]. Again, the implications of such an early increase are not immediately apparent, but they could serve as an early marker for prion infection, one which occurs many weeks prior to dendritic spine loss, and possibly at a time when therapeutic intervention could have a better chance of success. Using the open model system of POSCA, further investigation into these early changes is now possible.
